# Gosha-Jinki-Gan Recovers Spermatogenesis in Mice with Busulfan-Induced Aspermatogenesis

**DOI:** 10.3390/ijms19092606

**Published:** 2018-09-03

**Authors:** Ning Qu, Miyuki Kuramasu, Yoshie Hirayanagi, Kenta Nagahori, Shogo Hayashi, Yuki Ogawa, Hayato Terayama, Kaori Suyama, Munekazu Naito, Kou Sakabe, Masahiro Itoh

**Affiliations:** 1 Department of Anatomy, Division of Basic Medical Science, Tokai University School of Medicine, Kanagawa 259-1193, Japan; terahaya@tokai-u.jp (H.T.); suyama@is.icc.u-tokai.ac.jp (K.S.); sakabek@tokai-u.jp (K.S.); 2 Department of Anatomy, Tokyo Medical University, Tokyo 160-8402, Japan; kitaoka@tokyo-med.ac.jp (M.K.); anatomy@tokyo-med.ac.jp (Y.H.); kenta-n@tokyo-med.ac.jp (K.N.); sho5-884@umin.ac.jp (S.H.); yogawa@tokyo-med.ac.jp (Y.O.); itomasa@tokyo-med.ac.jp (M.I.); 3 Department of Anatomy, Aichi Medical University, 1-1 Yazakokarimata, Nagakute, Aichi 480-1195, Japan; munekazunaito@gmail.com

**Keywords:** oriental medicine, aspermatogenesis, testicular immunology, anticancer treatment

## Abstract

Busulfan is an anti-cancer chemotherapeutic drug and is often used as conditioning regimens prior to bone marrow transplant for treatment of chronic myelogenous leukemia. Male infertility, including spermatogenesis disturbance, is known to be one of the side effects of anticancer drugs. While hormone preparations and vitamin preparations are used for spermatogenesis disturbance, their therapeutic effects are low. Some traditional herbal medicines have been administered to improve spermatogenesis. In the present study, we administered Gosha-jinki-gan (TJ107; Tsumura Co., Ltd., Tokyo, Japan) to mice suffering from severe aspermatogenesis after busulfan treatment to determine whether TJ107 can recover spermatogenesis. Male 4-week-old C57BL/6J mice were administered a single intraperitoneal injection of busulfan, and they were then fed a normal diet for 60 days and then a TJ107 diet or TJ107-free normal diet for another 60 days. After busulfan treatment, the weight of the testes and the epididymal sperm count progressively decreased in the normal diet group. On the other hand, in the TJ107 group, these variables dramatically recovered at 120 days. These results suggest that busulfan-induced aspermatogenesis is irreversible if appropriate treatment is not administered. Supplementation of TJ107 can completely recover the injured seminiferous epithelium via normalization of the macrophage migration and reduction of the expressions of Tool-like receptor (TLR) 2 and TLR4, suggesting that TJ107 has a therapeutic effect on busulfan-induced aspermatogenesis.

## 1. Introduction

Busulfan (BSF; Myleran, 1,4-butanediol dimethanesulfonate) is an alkylating agent that primarily affects hematopoietic stem cells and results in bone marrow ablation. It is employed as an anti-cancer chemotherapeutic drug in childhood and adult chronic myelogenous leukemia before bone marrow transplantation. Previous studies have shown that BSF treatment at 100–150 mg/kg body weight is sufficient to induce myeloablation due to its toxic effect on these stem cells [1,2]. In the case of hematopoietic stem cells transplantation or graft-versus-host disease, the combination of BSF at 20–80 mg/kg/day for four days and cyclophosphamide at 100–200 mg/kg/day for two days is most commonly used for the thrombocytopenic effect and reduction in the number of leucocytes [3].

However, clinical treatment with BSF has been shown to cause gonadal dysfunction-amenorrhea and azoospermia in humans [4,5,6]. In mice, it has been demonstrated that a single dose of BSF destroyed differentiating spermatogonia and possibly stem cells—with the exception of a small subpopulation of cells attached to the basal membrane of the seminiferous epithelium [7,8,9,10,11]. Additionally, we found previously that at 60 days after BSF treatment (40 mg/kg), most germ cells were destroyed, and that not only the proliferative cells, but also the apoptosis-positive cells were strikingly reduced when compared with the findings in normal mice [12].

**Figure 1:**
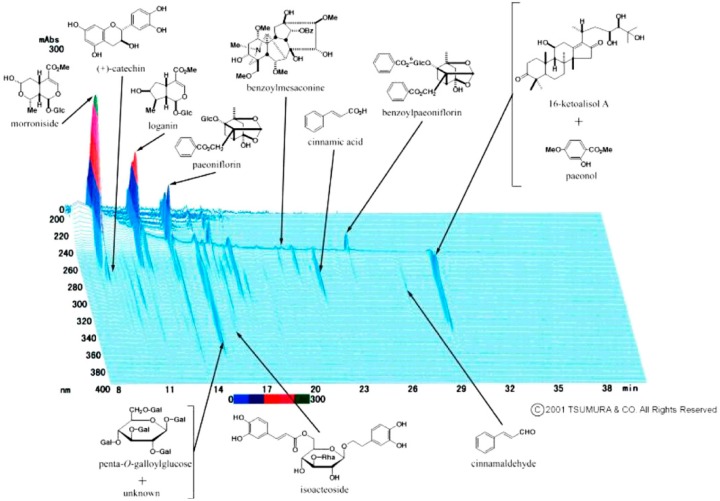
Three-dimensional high-performance liquid chromatography profile of Gosha-jinki-gan. Gal: galloyl.

Information on therapy for BSF-induced male infertility is limited. Recently, traditional herbal medicines have attracted attention and they are now used as complementary therapies in Western medicine such as a traditional Chinese medicine “Danggui Longui Wan”, which has potent activity against myelocytic leukemia [13], and “Qingyihuaji Formula”, which has significant anti-cancer effects in pancreatic cancer [14]. Generally, traditional herbal medicines are blended herbal drugs made of numerous crude components of natural origin to enhance the effects, and side effects are considered to be less frequent with these herbal drugs than with conventional therapies [15,16]. In Japan, traditional herbal medicines have been approved for clinical use by the National Health Insurance Program, and some traditional herbal medicines have been used in the treatment of male infertility. Gosha-jinki-gan, a traditional herbal medicine having 10 herbal components in fixed proportions, has been used to treat a variety of age-related conditions. Figure 1 shows the three-dimensional high-performance liquid chromatography profile of Gosha-jinki-gan (T107) reserved by Tsumura & Co. (Tokyo, Japan). The main indications for Gosha-jinki-gan in Japan are meralgia, lower back pain, numbness, and neuropathy in elderly patients, suggesting enhanced blood supply to nerves and the activation of an endogenous pain modulating system [17]. While several clinical studies have demonstrated the efficacy of Gosha-jinki-gan for the treatment of peripheral neuropathy induced by anticancer drugs [18,19,20,21], as well as diabetic neuropathy [22,23], to our knowledge, no study has assessed its possible therapeutic effect on infertility after anticancer treatment. Therefore, the present study aimed to determine whether Gosha-jinki-gan could recover spermatogenesis in BSF-induced aspermatogenesis.

## 2. Results

### 2.1. TJ107 Recovered Body Weight and Reproductive Parameters in BSF-Treated Mice

**Figure 2:**
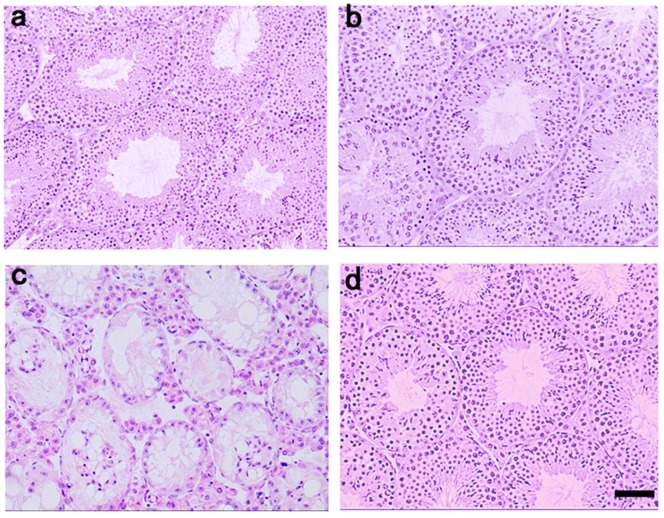
Histological examination of the testes (*n* = 10) at day 120 in each group. Intact seminiferous tubules showing all maturation stages of the germinal epithelium from spermatogonia to spermatozoa are seen in the control group (**a**) and the control+TJ107 group (**b**). Atrophic seminiferous tubules with azoospermia are seen in the BSF group (**c**). Normal-appearing seminiferous tubules are seen in the BSF+TJ107 group (**d**). Bar = 50 µm.

Table 1 summarizes body weight, and reproductive parameters in each experiment group after the treatment period. Body weight, absolute and relative testicular weights, and the epididymal spermatozoa count at day 60 were significantly lower in the BSF group than in the control group. At day 120, further decreases in all reproductive parameters were noted in the BSF group. Upon histological examination of the testes, atrophic seminiferous tubules with azoospermia were recognized (Figure 2c). In contrast, in the BSF+TJ107 group, body weight, absolute and relative testicular weights, the epididymal spermatozoa count, and the fertility rate showed dramatic recovery at day 120, although body weight and offspring numbers did not reach the values in the control group (Table 1). Moreover, testicular sections of the mice in the BSF+TJ107 group showed normal-appearing seminiferous tubules demonstrating all stages of maturation of the germinal epithelium from spermatogonia to spermatozoa (Figure 2d), similar with naive spermatogenesis in the control group (Figure 2a).

### 2.2. TJ107 Normalized Proliferation and Apoptosis in the Testes of BSF-Treated Mice

**Figure 3:**
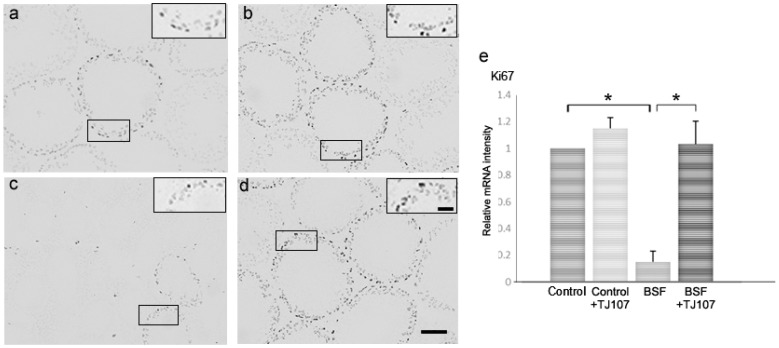
Detection of proliferating cells in the testes using antibodies against Ki67 nuclear antigen at day 120 in the control (**a**), control+TJ107 (**b**), BSF (**c**), and BSF+TJ107 groups (**d**). The enlarged part (Bar: 40 μm) of the seminiferous tubules is bounded by solid frames and is shown in the top right corner in each testis section. Ten testes from each group were examined. Dark brown spots indicating Ki67-positive nuclei of proliferating spermatogonia are detected in almost all seminiferous tubules in the control (**a**), control+TJ107 (**b**), and BSF+TJ107 (**d**) groups at day 120. Most germ cells are destroyed and few seminiferous tubules with Ki67-positive cells are sporadically observed in the BSF group (**c**). Bar: 40 μm. (**e**) Expression of Ki67 in testicular tissues of mice from each group (*n* = 10). Asterisks indicate *p* < 0.05.

**Figure 4:**
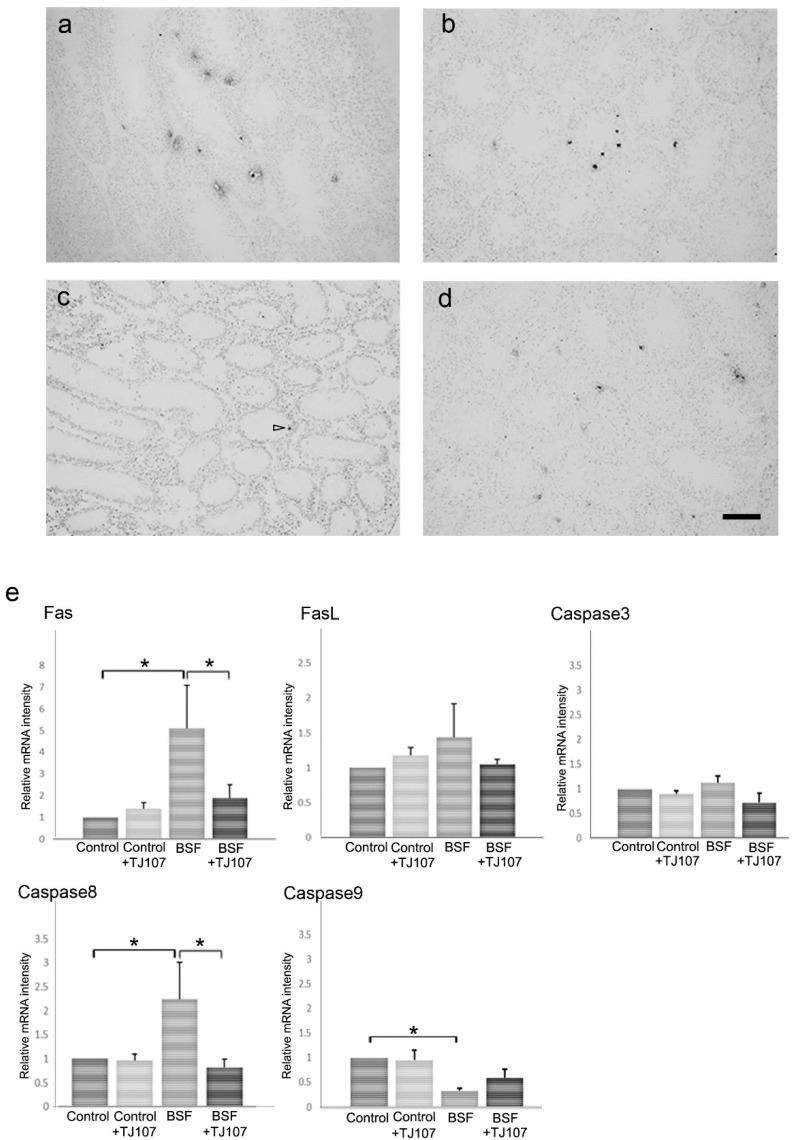
Histological detection of TUNEL staining in testicular sections of mice from the control (**a**), control+TJ107 (**b**), BSF (**c**), and BSF+TJ107 (**d**) groups at day 120. Ten testes from each group were examined. Dark brown spots indicating TUNEL-positive nuclei of apoptotic germ cells are seen. Some TUNEL-positive germ cells are detected in the seminiferous tubules in the control (**a**), control+TJ107 (**b**), and BSF+TJ107 (**d**) groups at day 120. Most germ cells are destroyed at day 120 in the BSF group; thus, TUNEL-positive cells (arrowhead) are hardly detected (**c**). Bar: 40 μm. (**e**) Expressions of Fas, FasL, Caspase3, Caspase8, and Caspase9 in testicular tissues of mice from each group (*n* = 10). Asterisks indicate *p* < 0.05.

We assessed proliferation and apoptosis in the testes of mice from each study group at day 120. In the BSF group, only a few seminiferous tubules with proliferating spermatogonia were detected (Figure 3c); however, apoptotic germ cells were hardly detected because most of the germ cells were destroyed (Figure 4c). In the BSF+TJ107 group, proliferating spermatogonia were detected in almost all the seminiferous tubules (Figure 3d), and some apoptotic germ cells were detected in the seminiferous tubules (Figure 4d), similar with the finding in the control group (Figure 4a). Additionally, the expressions of proliferation-related genes (*Ki67*) and apoptotic genes (*Fas*, *FasL*, *Caspase3*, *Caspase8*, and *Caspase9*) in the testicular tissues of mice from each group were examined. The expression of *Ki67* was significantly decreased only in the BSF group and not in the other three groups, and the expressions of *Fas* and *Caspase8* were significantly increased only in the BSF group and not in the other three groups (Figure 4e). The expression of *FasL* and *Caspase3* was not significantly increased in all the four groups.

### 2.3. TJ107 Suppressed Macrophage Migration via Reduction of the Expressions of TLR2 and TLR4 in BSF-Treated Mice

**Figure 5:**
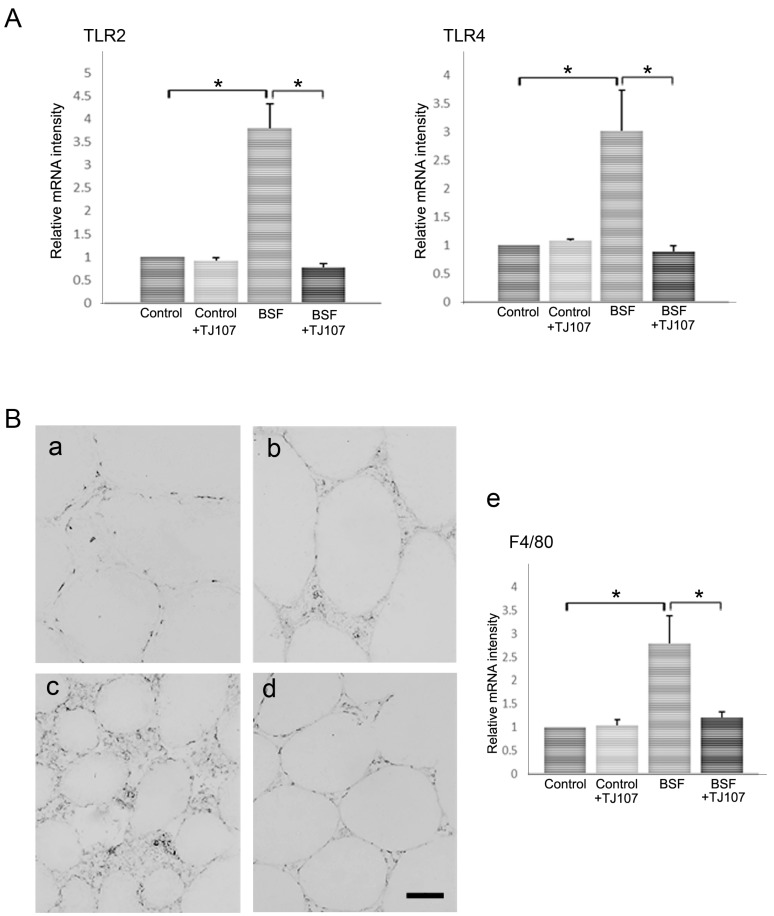
TLR expression and macrophage migration at day 120 in each group. **A**: Expressions of TLR2 and TLR4 in testicular tissues of mice from each group (*n* = 10). Asterisks indicate *p* < 0.05. **B**: F4/80 antigen-positive cells in testicular sections (*n* = 10) of mice from the control (**a**), control+TJ107 (**b**), BSF (**c**), and BSF+TJ107 (**d**) groups. Many macrophages are seen infiltrating the interstitium around the atrophic seminiferous tubules in the BSF group (**c**). Bar = 20 µm. (**e**) Expression of the macrophage marker gene in testicular tissues of mice from each group (*n* = 10). Asterisks indicate *p* < 0.05.

Considering that BSF-induced spermatogenic cell damage can upregulate TLR2 and TLR4 expressions in Sertoli cells and facilitate macrophage infiltration into the testes [23], we examined the effects of TJ107 on TLR expression and macrophage migration in the testes. The mRNA levels of TLR2, TLR4, and F4/80 were dramatically increased in the BSF group; however, these levels were normal in the BSF+TJ107 group (Figure 5A,B-e). In addition, we examined migratory macrophages in the testes of mice from each group according to immunohistochemistry targeting F4/80. Compared with the findings in the control group (Figure 5B-a), the increased macrophage migration induced by BSF (Figure 5B-c) was significantly inhibited by TJ107 (Figure 5B-d).

## 3. Discussion

We identified the therapeutic effect of TJ107 on BSF-induced infertility. Mice treated with BSF showed significant decreases in body weight, absolute and relative testes weights, the numbers of both proliferating spermatogonia and spermatozoa, and the fertility rate. This aspermatogenesis was irreversible for at least 360 days unless medication was administered (Qu, N., Tokyo Medical University, Tokyo, Japan. 2016; testes weights: 0.011 ± 0.007 g; epididymal spermatozoa: 0.089 ± 0.008 × 10^5^ cells). The BSF-treated mice that subsequently received TJ107 showed significant recovery in body weight, absolute and relative testes weights, the numbers of both proliferating spermatogonia and spermatozoa, and the fertility rate, and there was no significant macrophage infiltration in the testes. These results indicated that TJ107 could completely regenerate the seminiferous epithelium injured with BSF treatment by remedying the testicular immunology circumstance. To our knowledge, this is the first study to demonstrate that TJ107 has a therapeutic effect on infertility after anticancer treatment.

Infertility has become an important quality of life issue. Animal and human studies have indicated that chemoradiotherapy is mutagenic to germ cells at various stages of maturation [24]. Fertility preservation is becoming an emerging discipline owing to increased awareness over the last 20 years among researchers, clinicians, and patients regarding extended survival after cancer treatment [25]. Some trials have attempted to evaluate the use of gonadotropin-releasing hormone agonists or antagonists, or testosterone with or without estradiol or progesterone for restoring the capability of spermatogonia to differentiate and recover normal spermatogenesis [26]. However, there has been difficulty regarding clinical therapy for male infertility after chemotherapy treatment. The most common causes of male infertility include spermatogenesis disturbance, such as low sperm count and/or poor sperm quality [27]. In traditional herbal medicine, the term “kidney (Shen-qi in Chinese or Jin-ki in Japanese)” in the oriental theory not only refers to the kidney but also considers the material basis of human body growth, development, and reproduction [28]. Therefore, the concept of “kidney replenishing” is important for spermatogenesis. In other words, low sperm count and/or poor sperm quality is due to the loss of Shen-qi or Jin-ki. Gosha-jinki-gan (TJ107), a “kidney-replenishing” herbal medicine, has been recently demonstrated to be effective in the treatment of peripheral neuropathy induced by anticancer drugs [18,19,20,21]. In the present study, we found that Gosha-jinki-gan was effective for reproductive failure after anticancer treatment (Figure 2 and Table 1).

It is well known that spermatogenesis is a complex process and germ cell apoptosis is essential. Previous studies reported that several apoptotic pathways are activated during male germ cell differentiation in response to different alkylating agents such as cyclophosphamide and BSF [29,30]. During this time, we demonstrated the BSF-treated mice exhibited a marked increase in apoptosis and the reduced *Fas* and *Caspase8* gene expressions were detected in BSF+TJ107 group (Figure 4), indicating that the supplementation of TJ107 can avoid BSF-induced germ cell apoptosis via the *Fas/FasL* and *Caspase8* pathways. Concurrently, the number of Ki67-immunolabelled spermatogonia and testicular *Ki67* gene expression were higher in the mice from BSF+TJ107 group in comparison with BSF group, suggesting that TJ107 may be related to the spermatogonial proliferation. A previous study demonstrated that BSF-induced spermatogenic cell damage can upregulate TLR2 and TLR4 expressions in Sertoli cells and facilitate macrophage infiltration into the testes [31]. We also had the same results and showed that TJ107 could inhibit increases in TLR2 and TLR4 expressions and significant infiltration of macrophages in the testes after BSF treatment (Figure 5). While the above results are not interpreted as a completely causal connection, these data provide novel insights into the mechanisms underlying BSF-induced aspermatogenesis and stand a good chance of clinical therapy on infertility by chemotherapy.

It is notable that TJ107 not only histologically but also physiologically improved spermatogenesis after BSF treatment; however, the number of offspring was less than that in the control group. Additionally, TJ107 could not completely recover body weight to the level in the control group (Table 1). Previously, Dioscoreae rhizoma (*Dioscorea batatas* Decaisne), one of the components in TJ107, was reported in vitro and in vivo to induce the release of growth hormones in rats [32]. Therefore, TJ107 might be more specific to reproductive dysfunction than growth decline. In fact, we have administered TJ107 from the day of BSF-injected to 60 days after injection, a quantity of recovery in spermatogenesis was detected (Qu, N., Tokyo Medical University, Tokyo, Japan. 2016; Epididymal spermatozoa (×10^5^): 6.038 ± 0.987). The different recuperation effect is not clarified at the present moment and we will unravel the mechanism of the prophylaxis/therapy effect of TJ107 on mature and immature mice in the next study.

In future experiments, we will examine the effects of TJ107 on irradiation-induced spermatogenic disturbance and evaluate the efficacy of TJ107 for improving fertility in oncologic patients.

## 4. Materials and Methods

### 4.1. Animals

C57BL/6J male mice aged 4 weeks (weighting 16–20 g) were purchased from SLC (Shizuoka, Japan) and were maintained in the Laboratory Animal Center of Tokyo Medical University under a 12 h light–dark cycle at controlled temperature of 22–24 °C and relative humidity of 50–60%.

### 4.2. Preparation of a Gosha-Jinki-Gan Diet

Gosha-jinki-gan (TJ107) (extract granules in powdered form; No. 2110107010) was manufactured by Tsumura & Co. (Tokyo, Japan) according to Japanese and international manufacturing guidelines. TJ107 is a standardized prescription medicine containing 10 crude components: Rehmanniae radix 5 g; Achyranthis radix 3 g, Corni fructus 3 g, Dioscoreae rhizome 3 g, Plantaginis semen 3 g, Alismatis rhizome 3 g, Hoelen 3 g, Moutan cortex 3 g; Cinnamomi cortex 1 g, processed Aconite tuber 1 g. A TJ107 diet was prepared as a standard mouse diet (standard certified diet (MF diet); 23.1% crude protein [*w*/*w*], 5.1% crude fat, 5.8% crude ash, 2.8% crude fiber, and 55.3% nitrogen-free extract and mineral mixture) containing 5.4% (*w*/*w*) TJ107 extract by Oriental Yeast Co., Ltd. (Tokyo, Japan).

### 4.3. Experimental Design

BSF (Busulfan, Sigma, St. Louis, MO, USA) was first dissolved in dimethyl sulfoxide (DMSO; Sigma) and, then, 100 μL of distilled water (DW) was added to achieve a final concentration of 40 mg/kg.

**Figure 6:**
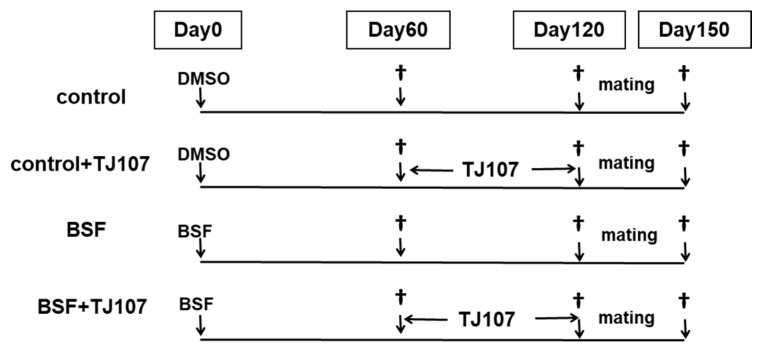
Time schedule of treatment applied to the mice in each group.

The study mice were randomly divided into the following four groups: Control group (*n* = 30; mice that received a single intraperitoneal injection of DMSO and were fed the standard MF diet to 120 days after injection); control+TJ107 group (*n* = 30; mice that received a single intraperitoneal injection of DMSO and were fed the standard MF diet to 60 days after injection and then the TJ107 diet to 120 days after injection); BSF group (*n* = 30; mice that received a single intraperitoneal injection of BSF and were fed the standard MF diet to 120 days after injection); and BSF+TJ107 group (*n* = 30; mice that received a single intraperitoneal injection of BSF and were fed the standard MF diet to 60 days after injection and then the TJ107 diet to 120 days after injection) (Figure 6). General condition, food intake, and body weight were documented for all the mice at 10-day intervals from 60 to 120 days after injection. All the experimental protocols in this study were carried out in accordance with the guidelines of the National Institutes of Health and were approved by the Tokyo Medical University Animal Care and Use Committee (approval code 27001 on 11 April 2015).

At 60 days after injection, DMSO-treated mice (*n* = 10) and BSF-treated mice (*n* = 10) were deeply anesthetized with pentobarbital (65 mg/kg body weight) and the testes were removed for gravimetry and epididymides were removed for epididymal spermatozoa count.

At 120 days after injection, to assess in vivo fertilization ability, we cross-mated mice from each group (*n* = 5) with normal C57BL/6J female mice (8–10 weeks of age; SLC, Shizuoka, Japan) at a male-to-female ratio of 1:2 in a cage for 1 month and determined the fertility rate for each group. At the same time, the remaining mice from each group (*n* = 20) were deeply anesthetized and the testes and epididymides were immediately removed for examination (Figure 6).

### 4.4. Histological Examination of the Testes

The testes of mice from each group (*n* = 5) were fixed with Bouin’s solution in whole and embedded in plastic (Technovit7100; Kulzer & Co., Wehrheim, Germany). Sections (5 μm) were obtained at 15–20-μm intervals and were stained with Gill’s hematoxylin and 2% eosin Y (Muto PC, Tokyo, Japan) for observation under light microscopy.

### 4.5. Histopathological Assessment

The testes of mice from each group (*n* = 5) were placed in OCT compound (Miles Laboratories, Naperville, IL, USA) and were stored at −80 °C until use. Sections of 6 µm were incubated with Block Ace (Yukijirushi, Hokkaido, Japan) for 20 min at room temperature (RT) and were then incubated for 2 h with rabbit anti-mouse Ki67 monoclonal antibodies (Abcam, Cambridge, United Kingdom, 1:1000 dilution) or rat anti-mouse F4/80 monoclonal antibodies (macrophages; Abcam, 1:100 dilution). After washing, the sections were incubated for 30 min with goat anti-rabbit or rabbit anti-rat IgG (Vector, Burlingame, CA, USA). Immunoreactive cells were visualized using Vectastain Elite ABC Reagent (Vector Laboratories, Burlingame, CA, USA) with 0.05% 3,3-diaminobenzidine-4HCl (DAB, Nickel Solution) and 0.01% H_2_O_2_ as the chromogen.

For the detection of apoptotic cells, TUNEL was performed according to the method described previously [33]. A commercially available kit (Apop Tag Plus Peroxidase In Situ Apoptosis Detection Kit; Serologicals Corporation, NY, USA) was used for the detection of the 3′-OH end of DNA. Deparaffinized sections (5–6 µm) of mice from each group (*n* = 5) were treated with proteinase K at 37 °C for 15 min. Endogenous peroxidase activity was blocked by treating the sections with 3% H_2_O_2_ in PBS for 5 min at RT. The sections were incubated with a mixture of terminal deoxynucleotidyl transferase (TdT) and digoxigenin-labeled dideoxynucleotide in a humidified chamber at 37 °C for 1 h. The sections were incubated with an anti-digoxigenin peroxidase conjugate for 30 min after a stop buffer for 10 min. Peroxidase activity was detected by exposing the sections to a solution containing 0.05% DAB.

### 4.6. Analysis of the mRNA Expressions of Cytokines Using Real-Time RT-PCR

The testes from mice in each group (*n* = 5) were examined. The iCycler thermal cycler (Bio-Rad, Hercules, CA, USA) was used to perform the PCR reactions, and the mixtures were stored at −80 °C until analysis. Real-time RT-PCR was performed on 3 ng cDNA using the validated SYBR Green gene expression assay in combination with SYBR Premix Ex Taq™ (TaKaRa, Bio Inc., Ohtsu, Japan) for measuring Ki67, Fas, FasL, Caspase3, Caspase8, Caspase9, TLR2, TLR4, F4/80, and GAPDH. Quantitative real-time PCR was performed in duplicate with the Thermal Cycler Dice Real-time System TP800 (TaKaRa). The Thermal Cycler Dice Real-time System software (TaKaRa) was used to analyze the data, and the comparative *C*_t_ method (2∆∆*C*t) was used to quantify gene expression levels. The results were expressed relative to the amount of GAPDH transcript used as an internal control. Relative mRNA intensity was calculated, and the expression in the control group for each point was normalized as one. The data are presented as the mean ± standard deviation. Table 2 lists all the primers used in this analysis.

### 4.7. Epididymal Spermatozoa Count

The number of epididymal spermatozoa were recovered from both epididymes of mice in each group (*n* = 20). Briefly, the epididymides were dissected out and cut into six pieces in PBS. All pieces were gently pipetted and then were passed through a stainless-steel mesh. The epididymal spermatozoa were harvested by centrifugation at 400 g for 10 min and were resuspended in 5 mL of PBS after washing thrice with PBS.

### 4.8. Statistical Analysis

ANOVA was used to analyze the differences. A *p*-value < 0.05 was considered statistically significant.

## 5. Conclusions

Aspermatogenesis after BSF treatment at 40 mg/kg is irreversible unless medication is administered. Supplementation with TJ107 can completely recover the injured seminiferous epithelium via normalization of macrophage migration and the reduction of the expressions of TLR2 and TLR4.

## Figures and Tables

**Table 1:** Testicular weights and epididymal spermatozoa numbers in the study groups.

Groups	Days after Injection	Control	Control+TJ107	BSF	BSF+TJ107
Body weight (g)	Day 60	28.301 ± 1.090	28.301 ± 1.090	25.913 ± 1.362 ^a^	25.913 ± 1.362 ^a^
	Day 120	34.010 ± 0.917	34.005 ± 1.240	27.862 ± 2.203 ^a^	31.008 ± 1.524 ^a,b^
Absolute testis weight (g)	Day 60	0.098 ± 0.002	0.098 ± 0.002	0.041 ± 0.008 ^a^	0.041 ± 0.008 ^a^
	Day 120	0.099 ± 0.002	0.102 ± 0.005	0.017 ± 0.002 ^a^	0.100 ± 0.006 ^b^
Relative testis weight (%)	Day 60	0.354 ± 0.013	0.354 ± 0.013	0.174 ± 0.017 ^a^	0.174 ± 0.017 ^a^
	Day 120	0.291 ± 0.004	0.303 ± 0.008 ^a^	0.060 ± 0.007 ^a^	0.334 ± 0.012 ^b^
Epididymal spermatozoa (×10^5^)	Day 60	19.800 ± 2.180	19.800 ± 2.180	1.575 ± 0.308 ^a^	1.575 ± 0.308 ^a^
	Day 120	20.500 ± 2.462	27.468 ± 1.578 ^a^	0.217 ± 0.019 ^a^	21.680 ± 1.700 ^b^
Fertility rate	Day 120	100% (10/10)	100% (10/10)	0% (0/10) ^a^	100% (10/10) ^b^
Number of fetuses/female	Day 150	5.875 ± 2.368	5.750 ± 1.639	0 ^a^	3.272 ± 2.014 ^a,b^

Data are presented as mean ± standard deviation. Relative testis weight was calculated in percentage by dividing the combined weight of both testes in milligrams by body weight in grams. ^a^ *p* < 0.05 vs. control group; and ^b^ *p* < 0.05 vs. BSF group.

**Table 2:** Primers used for real-time RT-PCR.

Gene	Forward Primer (5′-3′)	Reverse Primer (5′-3′)
*Caspase3*	GAGGCTGACTTCCTGTATGCTT	AACCACGACCCGTCCTTT
*Caspase8*	TTGAACAATGAGATCCCCAAA	CCATTTCTACAAAAATTTCAAGCAG
*Caspase9*	TGCAGTCCCTCCTTCTCAG	GCTTTTTCCGGAGGAAGTTAAA
*Fas*	GCAGACATGCTGTGGATCTGG	TCACAGCCAGGAGAATCGCAG
*FasL*	TCCAGGGTGGGTCTACTTACTAC	CCCTCTTACTTCTCCGTTAGGA
*F4/80*	CTTTGGCTATGGGCTTCCAGTC	GCAAGGAGGACAGAGTTTATCGTG
*Ki67*	GCTGTCCTCAAGACAATCATCA	GGCGTTATCCCAGGAGACT
*TLR2*	TGCAAGTACGAACTGGACTTCT	CCAGGTAGGTCTTGGTCATT
*TLR4*	GTGCCATCATTATGAGTGCC	CAAGCCAAGAAATATACCATCGAAG
*GAPDH*	TGTGTCCGTCGTGGATCTGA	TTGCTGTTGAAGTCGCAGGAG

## Abbreviations

BSF	Busulfan
TJ107	Gosha-jinki-gan
DMSO	Dimethyl sulfoxide
DW	Distilled water
